# Signal Transducer and Activator of Transcription (STAT) Proteins Regulate Mucosal‐Associated Invariant T (MAIT) Cell Function

**DOI:** 10.1111/imm.70086

**Published:** 2025-12-21

**Authors:** Olivia J. Cheng, Eimear K. Ryan, Michael Bennett, Christy Clutter, Jackson G. Cacioppo, Jeffrey Aubé, Andy E. Hogan, Daniel T. Leung

**Affiliations:** ^1^ Division of Microbiology & Immunology, Department of Pathology University of Utah Salt Lake City Utah USA; ^2^ Division of Infectious Disease, Department of Internal Medicine University of Utah Salt Lake City Utah USA; ^3^ Kathleen Lonsdale Institute for Human Health Research Maynooth University Maynooth Ireland; ^4^ Department of Chemistry University of North Carolina at Chapel Hill Chapel Hill North Carolina USA; ^5^ National Children's Research Centre Dublin Ireland

**Keywords:** Janus kinase/signal transducer and activator of transcription (JAK–STAT), metabolism, mucosal‐associated invariant T (MAIT) cells, regulation

## Abstract

Mucosal‐Associated Invariant T (MAIT) cells are a subset of T cells with potential for rapid cytotoxic and inflammatory functions. Dysregulation of the Janus Kinase‐Signal Transducer and Activator of Transcription (JAK–STAT) pathway, particularly involving STAT1 and STAT3, has been implicated in MAIT cell dysfunction in certain diseases. However, the transcriptional mechanisms regulating their effector functions, particularly the role of various STAT proteins, remain poorly understood. Using RNA sequencing and proteomics data, and experimental validation through in vitro assays using MAIT‐specific stimulation and small molecule inhibitors, we analysed the impact of STAT1, STAT3 and STAT5 on MAIT cell activation and function. Flow cytometric analysis was used to assess the functional implications of manipulating STAT proteins and the metabolic regulator HIF1α in MAIT cells. Our findings show that enhanced STAT1 activity negatively impacts MAIT cell effector functions, including granzyme B and interferon‐γ expression, while STAT3 and STAT5 are essential for promoting MAIT cell activation, function and glycolytic responses. Additionally, we identify HIF1α as a key regulator of these processes, suggesting that metabolic reprogramming plays a critical role in MAIT cell activation and function. This study highlights the critical roles of STAT1, STAT3, STAT5 and HIF1α in regulating MAIT cell effector functions, expanding our understanding of the molecular mechanisms underlying MAIT cell dysfunction. Our work lays the foundation for future research and applications aimed at modulating MAIT cell activity in immune‐related diseases and malignancies.

## Graphical abstract

Integrated transcriptomic, proteomic and flow cytometry analyses reveal that STAT1 negatively regulates MAIT cell effector and glycolytic functions, while STAT3, STAT5 and HIF1α act as positive modulators. Targeting these pathways may offer new strategies to restore MAIT cell function in immune dysregulation and cancer.

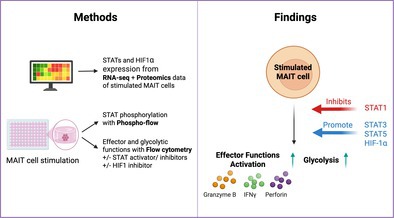

## 1 Introduction

Mucosal‐associated invariant T (MAIT) cells are an unconventional innate‐like subset of T cells that make up 1%–10% of T cells in the peripheral blood in humans and are abundant in mucosal sites such as the lungs and the gut [1, 2, 3, 4, 5, 6, 7]. MAIT cells express a semi‐invariant T cell receptor (TCR) that recognises nonpeptide microbial antigens presented on a nonclassical MHC‐Class I related molecule (MR1) [8, 9, 10, 11]. Various bacteria, including *Escherichia coli* , *Mycobacterium tuberculosis* and *Staphylococcus aureus* , as well as chemical ligands, such as 5‐(2‐oxopropylideneamino)‐D‐ribitylaminouracil (5‐OP‐RU), are known to activate MAIT cells [12, 13, 14]. MAIT cells also express cytokine receptors, such as IL‐12 and IL‐18 receptors, allowing for TCR‐independent activation by cytokines [15, 16, 17, 18, 19, 20]. Upon activation, MAIT cells display rapid effector functions, including the production of cytotoxic molecules granzyme B, perforin and proinflammatory cytokines such as interferon‐γ (IFN‐γ) [10, 17, 21].

MAIT cells participate in the immune response against a variety of diseases. They respond against bacterial infections, including antibiotic‐resistant bacteria, through the degranulation of cytolytic molecules [22, 23, 24]. Additionally, MAIT cells can respond to both acute and chronic viral infections, including human immunodeficiency virus (HIV) and severe acute respiratory syndrome coronavirus‐2 (SARS‐CoV‐2) [25, 26, 27]. In vitro, primary and chimeric antigen receptor (CAR) MAIT cells display cytotoxicity against cancer cell lines [28, 29]. Functional impairment of MAIT cells has been observed across a broad spectrum of diseases, including chronic infections, cancer and metabolic disorders like obesity, demonstrated by reduced levels of activation, degranulation and expression of effector molecules such as granzyme B and IFN‐γ [19, 30, 31, 32, 33, 34, 35]. However, the mechanisms leading to their activation and impairment are not well understood, prompting the need to investigate how their functions are inherently regulated under homeostatic conditions.

Transcriptional regulation plays a pivotal role in orchestrating the concerted programming of immune cells in response to external stimuli and signals. Transcription factors (TFs) serve as key facilitators, participating in signalling cascades, intricately coordinating the expression of genes to drive complex functional changes and adaptive responses. The Janus kinase‐signal transducer and activator of transcription (JAK–STAT) pathway is one of the major signalling pathways that convert and amplify external signals to a wide range of downstream biological events that regulate and modulate immune cell functions [36, 37, 38], and it has been associated with functional impairment in MAIT cells [34, 39]. Additionally, metabolic processes also play a crucial role in MAIT cell effector functions. The expression of granzyme B and IFN‐γ in MAIT cells is dependent on glycolysis [33, 40], and mTORC1 and MYC have been shown to regulate glycolysis in MAIT cells [33, 41]. Building on these observations, our objective is to examine the molecular mechanisms governing human MAIT cell effector functions.

## 2 Results

### 2.1 STATs Are Upregulated and Activated in MAIT Cells Upon Stimulation

**FIGURE 1:**
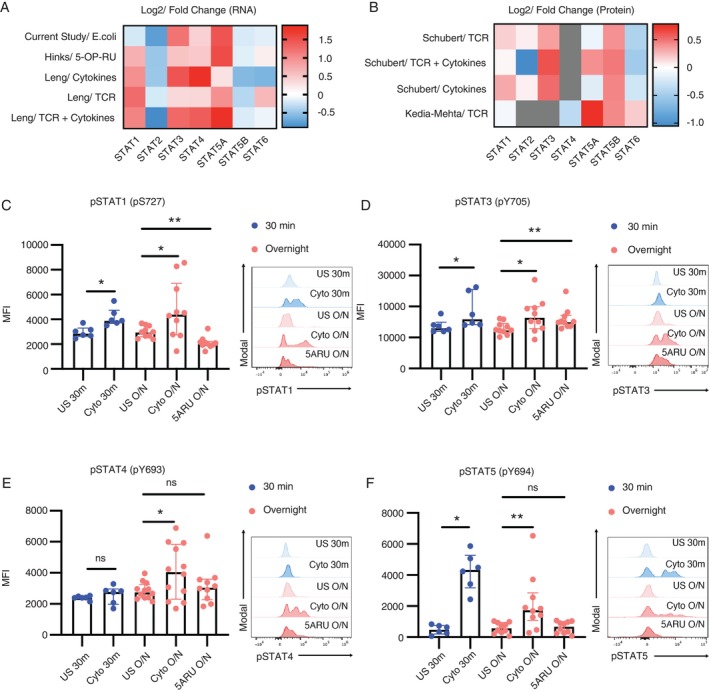
Changes in RNA and protein expression and phosphorylation levels of STATs in MAIT cells in various stimulation conditions compared to unstimulated controls. (A) Log2 fold change of STAT RNA expression in human MAIT cells upon stimulation, with data from the current study ( *Escherichia coli* stimulation) and publicly available data from Hinks et al. (5‐OP‐RU and cytokine stimulations) and Leng et al. (TCR and TCR with cytokine stimulations). (B) Log2 fold change of STATs protein expression in human MAIT cells upon TCR and TCR‐cytokines stimulations. Data were obtained from publicly available data from Shubert et al. and Kedia‐Mehta et al. Grey boxes indicate that data were not available. (C–F) PBMCs were stimulated for 30 min (blue) or overnight (pink), with IL‐12, IL‐15 and IL‐18 or 5‐A‐RU/MGO at 37°C and fixed at the end of the corresponding timepoints for phosphoflow analysis. The phosphorylation levels of (C) STAT1, (D) STAT3 (Y705), (E) STAT4 and (F) STAT5 (Y694) in MAIT cells were analysed, shown in median fluorescence intensity (MFI). Statistical analysis was performed using Wilcoxon signed‐rank test for paired unstimulated and stimulated samples of the same donors. *p < 0.05, ** *p* < 0.01. 30 m, 30‐min; Cyto, cytokines; O/N, overnight; US, unstimulated.

The STAT family of TFs is an important transcriptional regulator in immune responses [38, 42]. We first examined the gene expression of STATs in MAIT cells by performing bulk RNA sequencing (RNA‐seq) of human peripheral blood MAIT cells from healthy donors. We observed that STAT3, STAT4 and STAT5A RNA expression is higher in MAIT cells upon *E. coli* stimulation compared to unstimulated controls (Figure 1A). We then combined our data with publicly available RNA‐seq datasets of human MAIT cells stimulated by different methods, including with MAIT ligand 5‐OP‐RU, anti‐CD3/CD28 (TCR) and a combination of cytokines [43, 44]. We observed that STAT3, STAT4 and STAT5A are consistently upregulated upon various stimulations, while varied in magnitude of upregulation (Figure 1A). Consistently, at the protein level, publicly available proteomics datasets from Schubert et al. [45] and Kedia‐Mehta et al. [41] also revealed an increase in STAT3 and STAT5 protein expression in MAIT cells upon stimulation with TCR activation and/or cytokines (Figure 1B). Although STAT5B showed a modest decrease in RNA expression upon stimulation, this may reflect an increase in protein translation coupled with unchanged mRNA transcription (Figure 1). Together, these data suggested that upon stimulation, STAT3 and STAT5 are upregulated in MAIT cells at both the gene and protein levels.

Consistent with the bulk RNA‐seq findings, a publicly available single‐cell RNA sequencing (scRNA‐seq) dataset of MAIT cells [46] also shows that STAT1, STAT3, STAT4 and STAT5A are expressed transcriptionally in activated MAIT cells that express effector molecules (Figure S1A,B). Given the transient nature of STAT activities, which may not be fully captured by gene expression at the time of cell processing for sequencing, we analysed the inferred activity of each STAT TF using the R package decoupleR. We saw that all STATs except STAT6 were active primarily in cytokine‐stimulated MAIT cells (Figure S1B) and their activities overlapped with the expression of MAIT cell effector and activation markers (Figure S1A), suggesting that STATs are activated in stimulated MAIT cells.

To further confirm STAT functionality, we performed phosphoflow after 30‐min or overnight stimulation with cytokines, and overnight stimulation with the MAIT ligand 5‐Amino‐6‐(D‐ribitylamino)uracil with methylglyoxal (5‐A‐RU/MGO). Only overnight stimulation was used for 5‐A‐RU/MGO to allow sufficient time for MR1‐mediated antigen presentation. We observed that the expression levels of phosphorylated STAT3, STAT4 and STAT5 in MAIT cells increased upon stimulation with both 5‐A‐RU/MGO and cytokines (Figure 1C–F), suggesting that these 3 STATs are activated in response to both TCR‐dependent and ‐independent activations. Interestingly, STAT1 was upregulated only in cytokine‐stimulated but not MAIT ligand‐stimulated MAIT cells (Figure 1C). This differential activation suggests that STAT1 may play a more critical role in cytokine‐driven responses.

Together, these observations suggest that STAT3, STAT4 and STAT5 are involved in MAIT cell activation across different stimuli, while STAT1 activation is not induced upon MAIT ligand stimulation, indicating that STAT activation is selectively utilised depending on the stimulation context.

### 2.2 STAT1 Inhibits, While STAT3 and STAT5 Promote, MAIT Cell Functions

**FIGURE 2:**
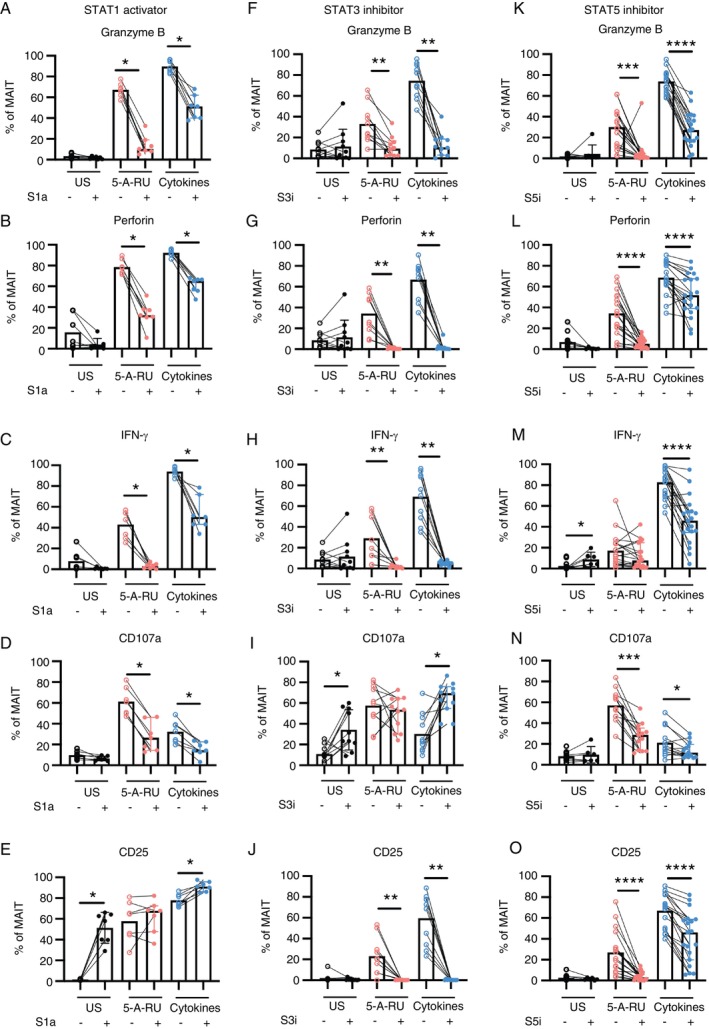
Expression of MAIT cell effector and activation markers in the presence of STAT modulators. PBMCs were pretreated with STAT modulators for 1 h and stimulated with 5‐A‐RU/MGO or cytokines IL‐12, IL‐15 and IL‐18 overnight. The expression of cytotoxicity and activation markers in MAIT cells in the presence of (A–E) STAT1 activator (S1a) 2‐N‐P, (F–J) STAT3 inhibitor (S3i) STATTIC and (K–O) STAT5 inhibitor (S5i) STAT5‐IN‐1. Statistical analysis was performed using the Wilcoxon signed‐rank test. * *p* < 0.05, **p < 0.01, ***p < 0.001. US, unstimulated.

To investigate the role of STAT proteins in regulating MAIT cell functions, we utilised STAT‐specific chemical modulators to alter their activity during both TCR‐dependent (5‐A‐RU/MGO) and TCR‐independent (IL‐12, IL‐15, IL‐18) in vitro stimulations. Previous studies have demonstrated that MAIT cells are dysfunctional in HIV infection, associated with an upregulation of STAT1 gene expression [34]. Based on this observation, we hypothesised that enhanced STAT1 signalling would impair MAIT cell effector functions. Using the selective STAT1 activator 2‐(1,8‐naphthyridin‐2‐yl)phenol (2‐NP) that prolongs the duration of STAT1 phosphorylation and enhances the transcription of STAT1 target genes [47], we observed reduced effector functions in MAIT cells. This impairment was evidenced by the decreased expression of the effector molecules granzyme B, perforin and IFN‐γ, as well as the degranulation marker CD107a in MAIT ligand‐ and cytokine‐stimulated MAIT cells in the presence of 2‐NP (Figure 2A–D). Interestingly, no impact was observed on the activation marker CD25 (Figure 2E). These findings suggest that prolonged and enhanced STAT1 activation may not affect the initial activation of MAIT cells but likely plays an important role in regulating the downstream expression and release of effector molecules post‐activation.

Given the increased expression and phosphorylation of STAT3 and STAT5 observed in stimulated MAIT cells (Figure 1), we used the small molecule inhibitors STATTIC (for STAT3) and STAT5‐IN‐1 (for STAT5), which prevent the dimerisation of these STATs, to evaluate their roles in MAIT cell regulation [48, 49]. STAT3 inhibition led to significantly reduced MAIT cell activation and effector functions in 5‐A‐RU/MGO and cytokine stimulations (Figure 2F–H,J). At a lower concentration (5 μM), the inhibitor tended to reduce degranulation levels, but this effect diminished with higher doses (Figure 2I), which was not observed in other markers (Figure S2). This highlights the importance of balanced STAT signalling, where STAT3 signalling amplitude differentially regulates effector molecules and degranulation. Similarly, the STAT5 inhibitor significantly reduced MAIT cell activation and effector functions (Figure 2K–O). Together, these data show that STAT3 and STAT5 are essential for MAIT cell activation and effector functions.

Since TCR‐dependent activation of MAIT cells, including via the 5‐A‐RU/MGO reaction, relies on MR1‐mediated antigen presentation, we explored the indirect effects of STAT1, STAT3 and STAT5 signalling on MAIT cells by examining their impact on MR1 expression in antigen‐presenting cells (APCs). In PBMCs stimulated with the MAIT ligand, MR1 expression was significantly lower in APCs (gated as CD45 + CD3‐HLADR+CD19‐) when treated with STAT3 and STAT5 inhibitors but not STAT1 activator (Figure S3). These results suggest that STAT3 and STAT5 may indirectly impact MAIT cell functions by modulating MR1 expression on APCs.

In summary, our data suggest that across both TCR‐dependent and TCR‐independent stimulations, prolonged STAT1 activity inhibits MAIT cell activation and effector functions, while STAT3 and STAT5 promote these responses. Notably, these TFs regulate MAIT cell function through both cell‐intrinsic mechanisms (as demonstrated by impaired response under cytokine‐driven activation) and cell‐extrinsic mechanisms (by modulating MR1 expression on APCs). These findings highlight the multifaceted roles of STAT signalling in shaping MAIT cell immunity.

### 2.3 HIF1α Is Essential But Not Sufficient for MAIT Cell Functions

JAK/STAT pathways are also regulated by environmental factors. For example, hypoxia induces phosphorylated STAT3 (pSTAT3) and the expression of the α subunit of hypoxia‐inducible factor‐1 (HIF‐1α) [50, 51]. Under normoxic conditions, cytokine‐activated STAT3 can interact and induce the upregulation of HIF1α proteins [50, 52]. HIF1α serves as a crucial element in hypoxia response and is a key regulator of glycolysis [53], a process essential for MAIT cell effector functions [33, 40]. Moreover, besides STAT signalling, engagement of the TCR/CD3 complex increases HIF‐1α protein synthesis [54, 55] and HIF‐1α regulates granzyme B expression in mice [56], highlighting its involvement in T cell immune regulation. Given its dynamic regulation in response to both TCR‐dependent and ‐independent activations, and its pivotal role in glycolysis, we aimed to investigate the specific role of HIF‐1α in regulating MAIT cell effector functions.

**FIGURE 3:**
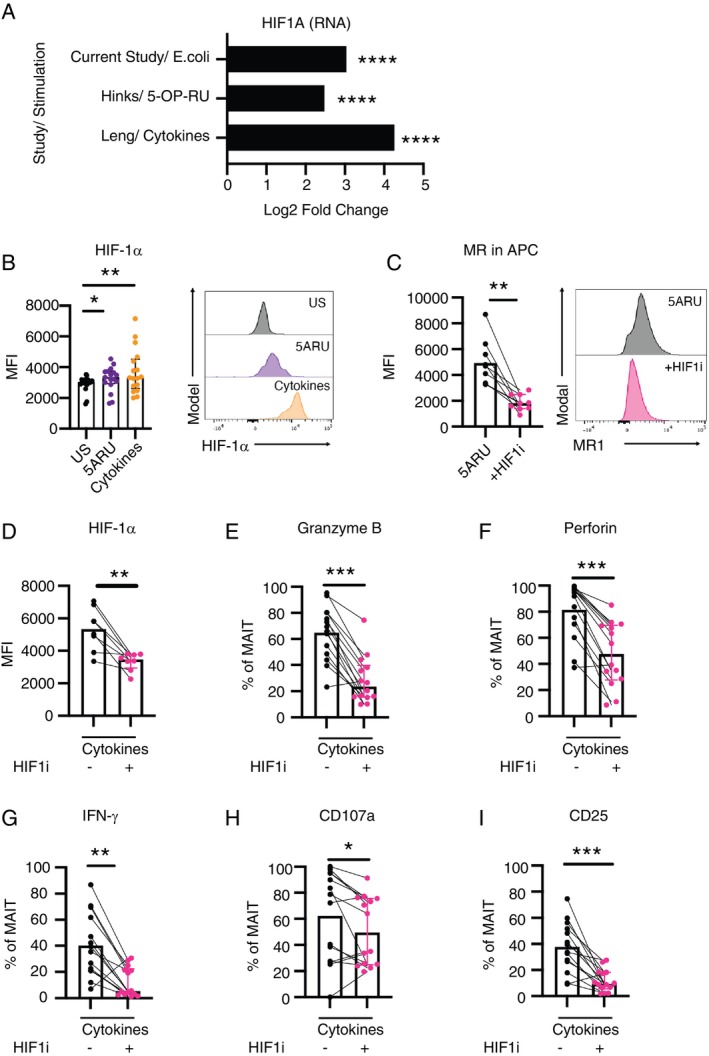
Expression of HIF‐1α in stimulated MAIT cells and the expression of MAIT cell effector and activation markers in the presence of HIF‐1α inhibitor. (A) Log2 fold change of HIF1A gene expression in MAIT cells from RNA‐seq data. (B) Flow cytometry analysis of HIF‐1α expression in stimulated MAIT cells. MAIT cells in PBMCs were stimulated overnight with 5‐A‐RU/MGO or cytokines IL‐12, IL‐15 and IL‐18. Expression of HIF‐1α was shown in median fluorescent intensity (MFI). (C) Expression of MR1 (in MFI) in APCs stimulated overnight with 5‐A‐RU/MGO. (D) Expression of HIF‐1α in cytokine‐stimulated MAIT cells with or without HIF‐1α inhibitor (HIF1i). (E–I) Expression of effector and activation markers in cytokine‐stimulated MAIT cells with or without HIF‐1α inhibitor. Flow cytometry data were collected from 2 or more independent experiments, and Wilcoxon‐rank test was used for statistical analysis. *p < 0.05, **p < 0.01, ***p < 0.001, ****p < 0.0001. APC, antigen presenting cells; US, unstimulated.

Based on the analysis of RNA‐seq datasets (our study, Hinks et al. [43], and Leng et al. [44]), along with flow cytometric data from our experiments on stimulated MAIT cells, we observed increased expression of HIF‐1α at both transcript and protein levels (Figure 3A,B). This finding is consistent with previous studies demonstrating that canonical HIF‐1α transcriptional targets—HK2, LDHA, GLUT1 and PKM2—are upregulated upon MAIT cell stimulation, further supporting HIF‐1α activation in this context [33, 41, 57, 58].

To determine if HIF‐1α regulates MAIT cell effector functions, we stimulated MAIT cells in PBMCs with cytokines, with or without HIF‐1α inhibitor PX‐478. Since STAT3 and STAT5 inhibitions led to significantly lower expression of MR1, we first assessed if HIF‐1α inhibition affects MR1 expression. Upon 5‐A‐RU stimulation, inhibition of HIF‐1α led to significantly reduced MR1 expression in APCs (Figure 3C). To focus on the intrinsic effect of HIF‐1α on MAIT cell function, cytokine stimulation but not 5‐A‐RU/MGO was used for the subsequent experiments. PX‐478 decreased the expression of HIF‐1α in stimulated MAIT cells (Figure 3D), and MAIT cell activation and cytotoxic function were impaired in the presence of the inhibitor (Figure 3E–I), suggesting that HIF‐1α plays an essential role in regulating MAIT cell functions. We were then interested to see if hypoxia, a condition that induces HIF‐1α expression, could enhance MAIT cell functions. We cultured PBMCs overnight in the presence of cobalt chloride II to chemically induce hypoxia. While such hypoxic condition led to a significant increase in HIF‐1α level (Figure S4A), we did not observe any enhancement in MAIT cell cytotoxic and effector functions (Figure S4B–D), suggesting that HIF‐1α, while essential for MAIT cell function, is not sufficient to enhance MAIT cell function during short‐term hypoxic conditions.

### 2.4 STAT3, STAT5 and HIF1α Modulate Glycolysis in MAIT Cells

Since glycolysis is crucial for MAIT cell effector functions [33, 40], we investigated the impact of STAT1, STAT3, STAT5 and HIF‐1α on glycolytic markers. Previous studies have reported that stimulation of MAIT cells leads to upregulation of key glycolytic enzymes, including Hexokinase‐II (HK2) and Pyruvate Kinase M2 (PKM2) [33, 58]. These enzymes are critical regulators of glycolytic activity, and prior functional studies demonstrated that the expression of HK2 and PKM2 affects glycolysis through Seahorse assays [41, 59, 60, 61].

**FIGURE 4:**
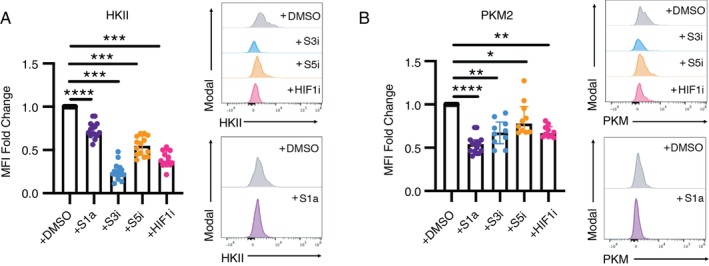
Expression of glycolysis markers in MAIT cells in the presence of STAT and HIF1α inhibitors. Glycolysis markers were analysed in MAIT cells using flow cytometry. Expression of (A) HKII and (B) PKM2 in cytokine‐stimulated MAIT cells. Data were collected from three independent experiments and were shown as MFI fold change compared to the solvent‐only, untreated condition. Wilcoxon signed‐rank test was used for statistical analysis. *p < 0.05, **p < 0.01, ***p < 0.001. HIF1i, HIF1 inhibitor; S1a, STAT1 activator; S3i, STAT3 inhibitor; S5i, STAT5 inhibitor.

In our experiments, inhibition of STAT3, STAT5 and HIF‐1α resulted in reduced expression of HK2 and PKM2 (Figure 4). Activation of STAT1 similarly led to decreased expression of these enzymes (Figure 4). These findings suggest that STAT3, STAT5 and HIF‐1α play important roles in positively regulating the expression of key glycolytic enzymes, while STAT1 may function as a negative regulator. Collectively, these data indicate that modulation of glycolytic pathways may be one of the mechanisms through which these TFs regulate MAIT cell effector functions.

### 2.5 T‐Bet Is Regulated by STAT Proteins and HIF1α, But Is Not Essential for theExpression of MAIT Cell Effector Molecules

Given our findings that STAT1, STAT3, STAT5 and HIF1α regulate MAIT cell effector functions, we explored the potential downstream TFs involved. One such candidate was T‐box TF 21 (T‐bet), a known regulator of effector functions in conventional T cells [62]. A previous study has shown that T‐bet expression correlates with the expression of granzyme B in MAIT cells [23], suggesting a potential regulatory effect of T‐bet on MAIT cell function. Therefore, we assessed the impact of STAT1, STAT3, STAT5 and HIF1α on T‐bet.

**FIGURE 5:**
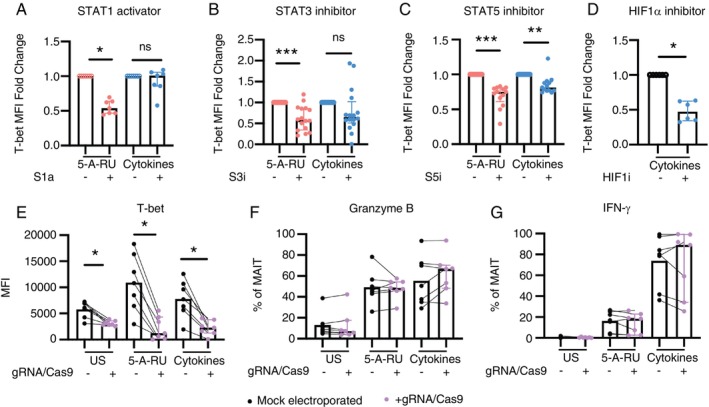
Expression of T‐bet in stimulated MAIT cells in the presence of STAT and HIF‐1α modulators. PBMCs were stimulated with 5‐A‐RU/MGO or cytokines. The expression of T‐bet with or without (A) STAT1 activator, (B) STAT3 inhibitor, (C) STAT5 inhibitor and (D) HIF‐1α inhibitor was analysed using flow cytometry and was expressed in fold change in MFI compared to untreated control. Expression of (E) T‐bet, (F) granzyme B and (G) IFN‐γ in MAIT cells with T‐bet knockout (purple) compared to control (black). Wilcoxon signed‐rank test was used for statistical analysis. *p < 0.05, **p < 0.01, ***p < 0.001. US, unstimulated.

In 5‐A‐RU/MGO stimulated MAIT cells, T‐bet expression was significantly reduced by all three STAT modulators (Figure 5A–C). In cytokine‐stimulated MAIT cells, a significant reduction of T‐bet expression was observed with STAT5 and HIF‐1α inhibitors (Figure 5C,D). These data suggest that in stimulated MAIT cells, STAT1, STAT3, STAT5 and HIF1α modulate T‐bet expression.

We were then interested in the effect of T‐bet on MAIT cell effector functions. We used CRISPR‐Cas9 to knock out T‐bet in MAIT cells. Despite successfully reducing T‐bet expression (Figure 5E), there was no impact on their effector functions, as indicated by the unchanged expression of granzyme B and IFN‐γ upon stimulation (Figure 5F,G). These results suggest that T‐bet is not essential for the expression of these effector molecules in MAIT cells.

## 3 Discussion

In this study, we investigated the roles of STAT1, STAT3, STAT5 and HIF‐1α in MAIT cell effector functions, contributing to the ongoing efforts in understanding the underlying transcriptional mechanisms. Our findings revealed the contrasting roles of these TFs—while enhanced STAT1 activity adversely affected MAIT cell effector functions, STAT3, STAT5 and HIF‐1α were crucial for promoting both activation and glycolytic responses in MAIT cells upon stimulation.

We showed that prolonged and enhanced STAT1 activity impaired MAIT cell effector functions without affecting activation. This observation aligns with the noted association between MAIT cell dysfunction and increased STAT1 gene expression in HIV infection [30, 34]. A potential mechanism for this impairment by augmented STAT1 activity could be interference with STAT3 activity and signalling [63], which we also showed to be essential for MAIT cell activation and function. Moreover, persons with gain‐of‐function (GOF) STAT1 mutations are more susceptible to bacterial infections [64]. However, the precise mechanism requires further examination in MAIT cells, underscoring the importance of investigating STAT1 hyper‐activation in MAIT cells in the context of various disease contexts.

STAT3 and STAT5 were indispensable for MAIT cell activation and the expression of effector molecules, highlighting their therapeutic potential in modulating MAIT cell‐mediated functions. The impact of STAT3 inhibition on the expression of granzyme B, perforin and IFN‐γ aligns with previous observations indicating that STAT3 may impair IL‐17A production [39], thereby further expanding our understanding of STAT3's role in regulating MAIT cell function. Similar to the effect of STAT1 on STAT3 mentioned above, STAT3 is also capable of suppressing STAT1 DNA‐binding activity through sequestration of STAT1 [65], though further validation of this reciprocal regulation in MAIT cells is warranted. Our results on STAT5 are also in agreement with previous studies that revealed its involvement in regulating MYC, a TF essential for MAIT cell proliferation [41], and in MAIT cell activation and effector response to IL‐7 and IL‐15 [18, 66]. Expanding beyond effector functions, we provide additional insights into the potential involvement of glycolysis in STAT5‐mediated regulation of MAIT cell function. Despite an upregulation of STAT4 in activated MAIT cells, we were unable to examine the inhibition of STAT4, as lisofylline, a STAT4 inhibitor, has an effective concentration that exceeded the tolerable DMSO level in MAIT cell culture. Similarly, while gain‐of‐function studies for STAT3 and STAT5 would provide valuable insights into their positive regulatory roles in MAIT cell function, the lack of specific pharmacological activators with selective activity limits our ability to perform such experiments.

Our study identified HIF‐1α as a critical modulator of effector functions and glycolytic enzymes in MAIT cells, aligning with previous findings demonstrating a dependency on glycolysis for MAIT cell effector functions [33, 40]. Molecularly, mTORC1 has been shown to regulate MAIT cell IFN‐γ expression and glycolysis [33], and also regulates HIF‐1α [55]. Our findings not only align with but also further expand the current understanding of the metabolic regulation underlying MAIT cell effector functions. Although we did not directly examine whether STAT1, STAT3 or STAT5 modulate HIF‐1α protein, future studies exploring the regulatory relationships among STATs, HIF‐1α and glycolysis will be important for establishing a more integrated mechanistic framework.

While our CRISPR‐Cas9 experiment demonstrated that T‐bet is dispensable for the expression of granzyme B and IFN‐γ in stimulated MAIT cells, it is important to note that a low level of T‐bet expression remained after gene editing. This residual T‐bet may still support these functions, and therefore, we cannot fully exclude its contribution. Additionally, it did not preclude the possibility that T‐bet regulates other aspects of MAIT cell functions or that there are redundant TFs involved.

MAIT cells exhibit altered functions in various diseases, such as acute and chronic viral infections, bacterial infections, metabolic diseases, autoimmune disease and cancer [32, 33, 34, 67, 68, 69, 70, 71, 72, 73]. Investigating the transcriptional signalling pathways in MAIT cells within these contexts could provide valuable insights into their pathophysiological roles and the potential in correcting their altered function. While our findings support a functional role for STAT1, STAT3, STAT5 and HIF‐1α in MAIT cell activation and metabolism, we acknowledge that confirming the physiological relevance of these pathways will require further in vivo studies. In particular, using disease‐relevant mouse models will be crucial to validate the contribution of these pathways to MAIT cell function under physiologic and pathologic conditions. Future work should also aim to identify direct transcriptional targets and additional regulatory factors downstream of these signalling nodes. Moreover, the potential for STAT and HIF‐1α activity to serve as biomarkers of MAIT cell dysfunction in disease settings remains an intriguing avenue for exploration.

In conclusion, our study established the adverse effect of enhanced STAT1 activity on MAIT cell effector functions, while highlighting the essential roles of STAT3, STAT5 and HIF‐1α in MAIT cell activation and the expression of glycolytic enzymes. These findings complement and expand our current understanding of the molecular regulation underlying MAIT cell activation and effector functions. By advancing the comprehension of the molecular regulation of MAIT cell effector functions, this study highlights the importance of investigating these pathways as potential targets in various disease contexts, laying the groundwork for developing immunotherapies aimed at modulating MAIT cell function.

## 4 Methods and Materials

### 4.1 Human PBMC Isolation and Stimulation

Human PBMCs were isolated from anonymous healthy blood donors obtained from the Associated Regional and University Pathologists Inc. (ARUP) blood bank using Ficoll‐Paque PREMIUM density gradient media (Cytiva Marlborough, Massachusetts, USA) and stored at −80°C. Cells were cultured in complete RPMI media (Gibco, Thermo Fisher Scientific, Waltham, MA, USA) supplemented with 10% Fetal bovine serum, 1X HEPES and 1X Penicillin/Streptomycin. To stimulate MAIT cells in PBMC, 5‐A‐RU (50 nM) and Methylglyoxal (50 μM, Sigma Aldrich, St. Louis, MO, USA) or a combination of cytokines with IL‐12 (Peprotech), IL‐15 (Peprotech), IL‐18 (MBL International Corporation) (50 ng/mL of each). For experiments using modulators, cells were pretreated with 200 uM 2‐NP (Cayman Chemical), 20 uM (unless stated otherwise) of STATTIC (Selleck Chemicals), 200 uM of STAT5‐IN‐1 (Selleck Chemicals) or 100 uM PX‐478 (Cayman Chemical) for 1 h before stimulation. Concentrations of 2‐NP were selected based on in‐house titration experiments to determine the optimal dose for MAIT cell responses (Figure S5). STATTIC [74], STAT5‐IN‐1 [18] and PX‐478 [75] concentrations were used as previously reported in the literature. An equal volume of DMSO was added to untreated conditions. Cultures were incubated overnight unless otherwise stated. Viability of MAIT cells with 2‐NP, STATTIC, STAT5‐IN‐1 and PX‐478 is shown in Figure S5. To induce hypoxia, cobalt chloride 0.1 M solution (Sigma Aldrich) was added to PBMCs at 100 uM 1 h before stimulation.

### 4.2 RNA‐seq Analysis

Vα7.2^+^ (TRAV1‐2^+^) cells were first magnetically sorted from PBMCs were incubated with THP‐1 cells overnight with or without *E. coli* strain BL21. Then, MAIT cells, defined as CD3+ CD4‐ Va7.2+ CD161+ cells, were sorted by a FACSAria II instrument (BD Biosciences). RNA was extracted using miRNeasy mini kit (Qiagen), and RNA‐seq was performed using the Illumina Hi‐Seq platform. Publicly available bulk RNA‐seq and scRNA seq datasets of MAIT cells were downloaded from the Gene Expression Omnibus (GEO) under accession numbers GSE123805 (Hinks et al. [43]), GSE129906 (Leng et al. [44]) and GSE194189 (Garner et al. [46]). For bulk RNA seq analysis, Fastq files were trimmed using TrimGalore (0.6.10), aligned to the human genome (Homo_sapiens.GRCh38.dna.primary_assembly.fa.gz, release 109) [76] using STAR (2.5.2b) [77] and feature counts were obtained using featureCounts under the package subread (2.0.1) [78]. Differential expression analysis was performed with DESeq2 (1.40.2) [79] to compute log2 fold changes.

For single‐cell RNA‐seq analysis, publicly available datasets of stimulated MAIT cells from GEO accession number GSE194189 (Garner et al.) using Seurat (V5.0.3) [80], following the developer's vignette ‘Seurat—Guided Clustering Tutorial’. Processed expression matrices were normalised via log transformation. The top 2000 most highly variable features were selected for further analysis, and data scaling was performed. Principal Component Analysis (PCA) was used for dimensionality reduction, with the first 20 components selected based on the Elbow Plot method. Uniform Manifold Approximation and Projection (UMAP) was used to visualise data. Clustering of cells was performed using the Louvain algorithm to identify distinct cell populations based on shared nearest neighbours.

### 4.3 Proteomics Data

Publicly available proteomics datasets of MAIT cells were downloaded from the supplemental materials of Schubert et al. [45] and Kedia‐Mehta et al. [41].

### 4.4 TF Activity Inference With decoupleR

TF activity inference was conducted using the Bioconductor package decoupleR (2.6.0) [81] according to the developer's vignette ‘Transcription factor activity inference from scRNA‐seq’. This analysis was performed on the Seurat object derived from the Garner et al. dataset mentioned above. Briefly, a curated collection of human TFs and their target genes was obtained from OmniPath. Normalised log‐transformed counts were extracted from the Seurat object, and a univariate linear model (ULM) was used to infer TF enrichment scores. The activity of each TF is plotted with UMAP reduction.

### 4.5 Phospho‐Flow

In a 96‐well plate, 2 × 10^6^ PBMCs were serum‐starved for 1–2 h before stimulation. PBMCs were stimulated with 5‐A‐RU (50 nM) and Methylglyoxal (B50uM, Sigma Aldrich, St. Louis, MO, USA) or a combination of cytokines with of IL‐12 (Peprotech), IL‐15 (Peprotech), IL‐18 (MBL International Corporation) (50 ng/mL of each) for 30 min or overnight. Ghost Dye Violet 510 (Tonbo) and MR1‐5‐OP‐RU (NIH Tetramer Core) were added to the cultures at the beginning (for 30‐min incubation) or at the last 30 min of incubation. To stop the reaction and fix cells, equal volume of prewarmed fixation buffer (Biolegend) was added to the cultures for 30 min at 37°C. Cells were washed once with FACS buffer at 400 g for 3 min. Cells were then permeabilised with 250 μL of prechilled True‐Phos Perm Buffer (Biolegend) at −20°C for 1 h. Cells were washed twice with FACS buffer and centrifuged at 1000 g at room temperature for 5 min. To stain for surface and intracellular markers, cells were stained with antibodies in FACS buffer and Brilliant Stain buffer Plus (BD Biosciences) for 45 min at room temperature. Cells were washed once and resuspended in fresh FACS buffer for analysis on an Aurora (Cytek Biosciences). All antibodies used were listed in Table S1. MAIT cells were gated as Lymphocytes, Live, CD45^+^, CD3^+^, MR1‐5‐OP‐RU^+^, Vα7.2^+^ and APCs were gated as All Cells, Live, CD45^+^, CD3^−^, HLA‐DR^+^, CD19^−^ (Figure S6). All paired unstimulated and stimulated samples from each donor were processed on the same day, using identical antibody cocktails, staining protocols and flow cytometry instrument settings and acquired in a single session per experiment to minimise batch effects and interexperiment variability.

### 4.6 Flow Cytometry

Cells were washed with PBS and stained with Zombie UV (Biolegend) for 20 min. For surface marker staining, cells were stained with antibodies and MR1‐5‐OP‐RU (NIH Tetramer Core) in FACS buffer (1X PBS with 2% FBS) and Brilliant Stain Buffer Plus (BD Biosciences) for 30 min. For intracellular staining, Brefeldin A (Biolegend) was added to each well 4 h before staining. Cells were fixed and permeabilised using Foxp3/Transcription Factor Staining Buffer Set (eBioscience, Thermo Fisher) according to the manufacturer's protocol. Cells were resuspended in fresh FACS buffer for analysis on an Aurora (Cytek Biosciences). All antibodies used were listed in Tables S2–S4. All paired control and inhibitor‐treated samples from each donor were processed on the same day using identical antibody cocktails, staining protocols and flow cytometry instrument settings and acquired in a single session per experiment to minimise batch effects and interexperiment variability.

### 4.7 CRISPR‐Cas9 Knockout of T‐Bet

The CRISPR‐Cas9 protocol was adapted based on methods from a previous publication [82], as well as protocols from IDT and STEM Cell Technologies.

Total T cells were enriched from PBMCs using MojoSort Human CD3 T Cell Isolation Kit (Biolegend). Isolated total T cells were activated on Day 0 using 25 uL/mL of a‐CD3/a‐CD28 T cell Activator (STEM Cell Technologies) in Immunocult T cell expansion medium (STEM Cell Technologies) supplemented with 8% SR serum replacement (Gibco, Thermofisher) and IL‐2 (Peprotech) (100 ng/mL) for 72 h. On Day 4, CRISPR ribonuclear protein (RNP) was prepared by complexing IDT predesigned guide RNA (gRNA) for TBX21/T‐bet and Alt‐R Cas9 nuclease V3 (IDT). Activated T cells were electroporated (1400 V, 30 ms, 1 pulse) with RNP, single‐stranded oligodeoxynucleotide (ssODN) for homology‐directed repair, and electroporation enhancer (IDT) using the Neon Electroporation System 10 μL kit (Invitrogen, Thermofisher). Mock electroporated cells were used as controls. Electroporated cells were allowed to recover in T cell expansion medium supplemented with IL‐2 for 7 days, followed by stimulation with 5‐A‐RU/MGO or cytokines. Protein levels of T‐bet in MAIT cells were measured using flow cytometry as mentioned above.

gRNA: 5′‐GATTAAACTTGGACCACAAC‐3′. ssODN: 5′/AlT‐R‐HDR1/G*T*GTCGGGGAAACTGAGGGTCGCGCTCAACAACCACCTGTAGATGACTAGTGGTCCAAGTTTAATCAGCACCAGACAGAGATGATCATCACCAAGCAGGGACGGTGAGTGCGGCGCGCCGGCCCTTGGGGCCTCTG*T*G/AlT‐R‐HDR2/3′.

### 4.8 Statistical Analysis

Statistical analysis was performed using GraphPad Prism version 8 for Mac (GraphPad Software). The Wilcoxon signed‐rank test was used for comparisons between paired samples from the same donor (e.g., unstimulated vs. stimulated conditions or treatment groups).

## Funding

This work was supported by the National Institutes of Health (R01AI130378, R21CA280224).

## Conflicts of Interest

The authors declare no conflicts of interest.

## Supporting information


**Data S1:** imm70086‐sup‐0001‐Supinfo.docx.

## Data Availability

The data that support the findings of this study are available from the corresponding author upon reasonable request.
