# Supplementary material for: Signal Transducer and Activator of Transcription (STAT) Proteins Regulate Mucosal‐Associated Invariant T (MAIT) Cell Function
Source: Immunology. 2025 Dec 21;178(1):97–108. doi: 10.1111/imm.70086 (PMC13079253; doi:10.1111/imm.70086)
Supplement: Supplementary file 1 — Data S1: imm70086‐sup‐0001‐Supinfo.docx. [file IMM-178-97-s001.docx]

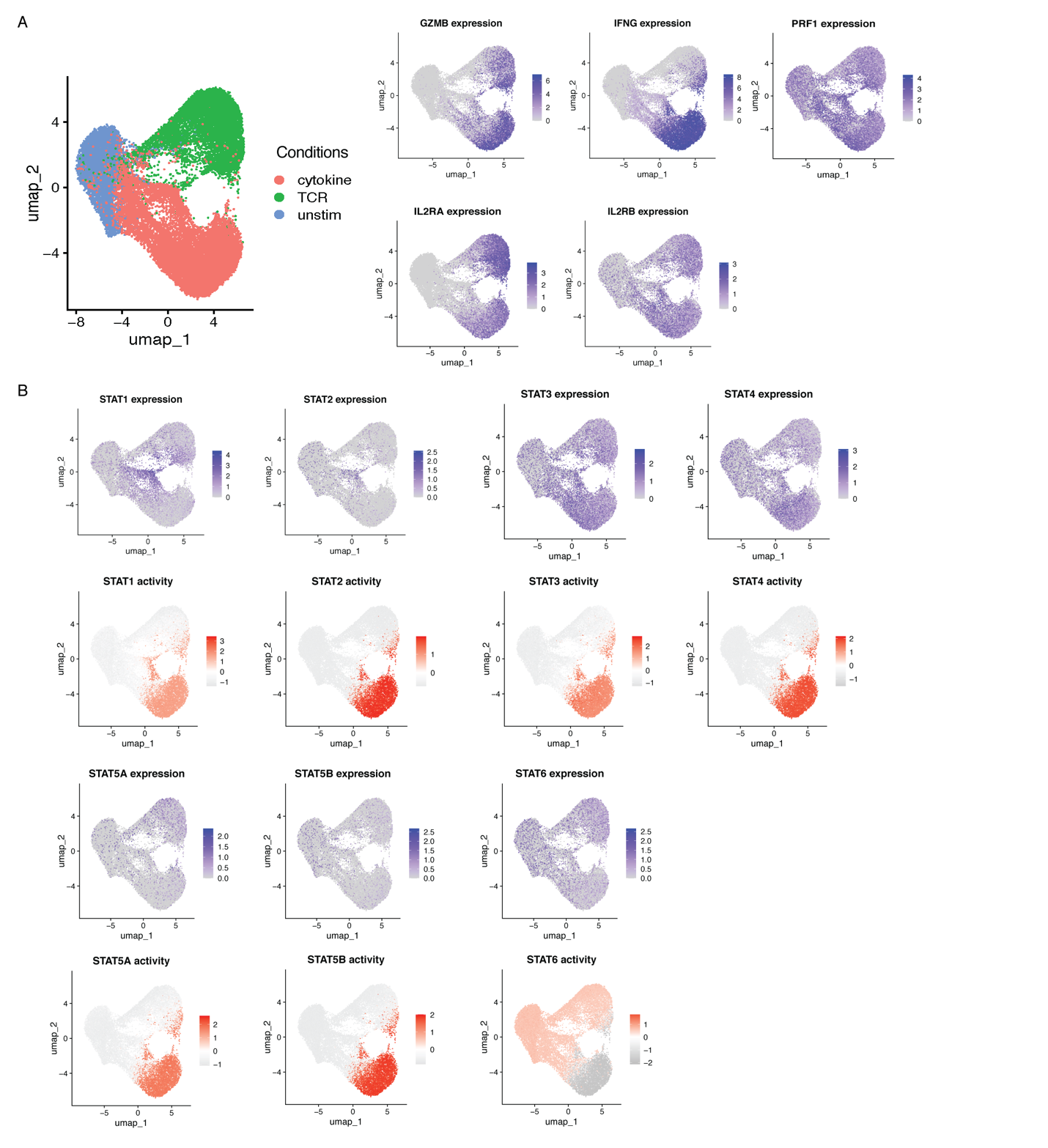


**Supplemental Figure 1. Inferred activities of STAT transcription factors in MAIT cells using publicly available single cell RNA sequencing data.**

A) UMAPs of human MAIT cells in 3 conditions: unstimulated, cytokine-stimulated, and TCR-stimulated and the expression of MAIT cell effector and activation markers. B) UMAPs showing the expression of each STAT and their corresponding inferred activities.


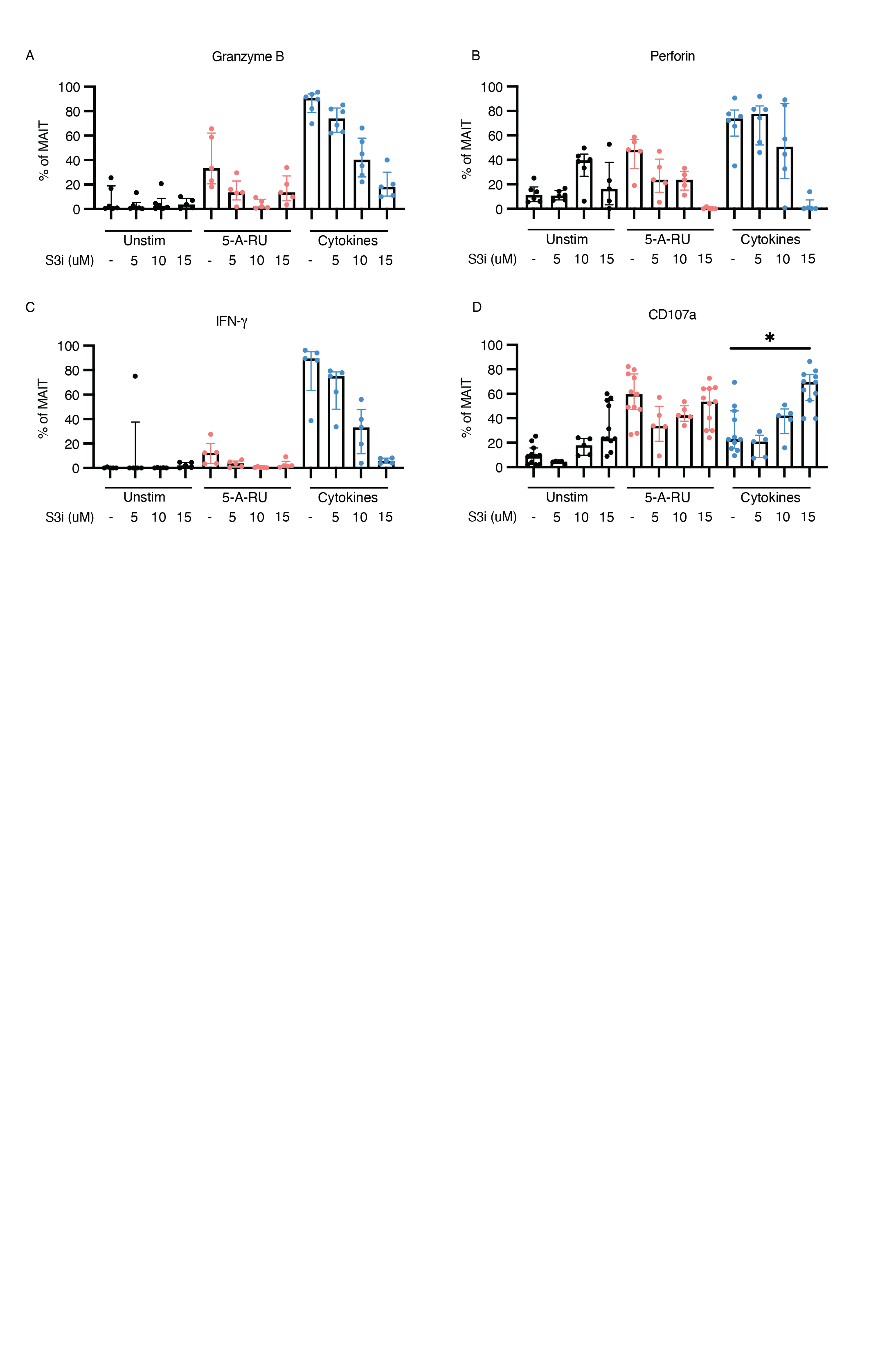


**Supplemental Figure 2. Expression of MAIT cell effectors and activation markers across various concentrations of STAT3 inhibitor.**

PBMCs were pretreated STAT3 inhibitor STATTIC for 1 hour and stimulated with 5-A-RU/MGO or cytokines IL-12, IL-15, and IL-18 overnight. The expression of cytotoxicity and activation markers in MAIT cells A) granzyme, B) perforin, C) IFN- γ, and D) CD107a, were analyzed using flow cytometry. Statistical analysis was performed using Wilcoxon signed-rank test. * p < 0.05.


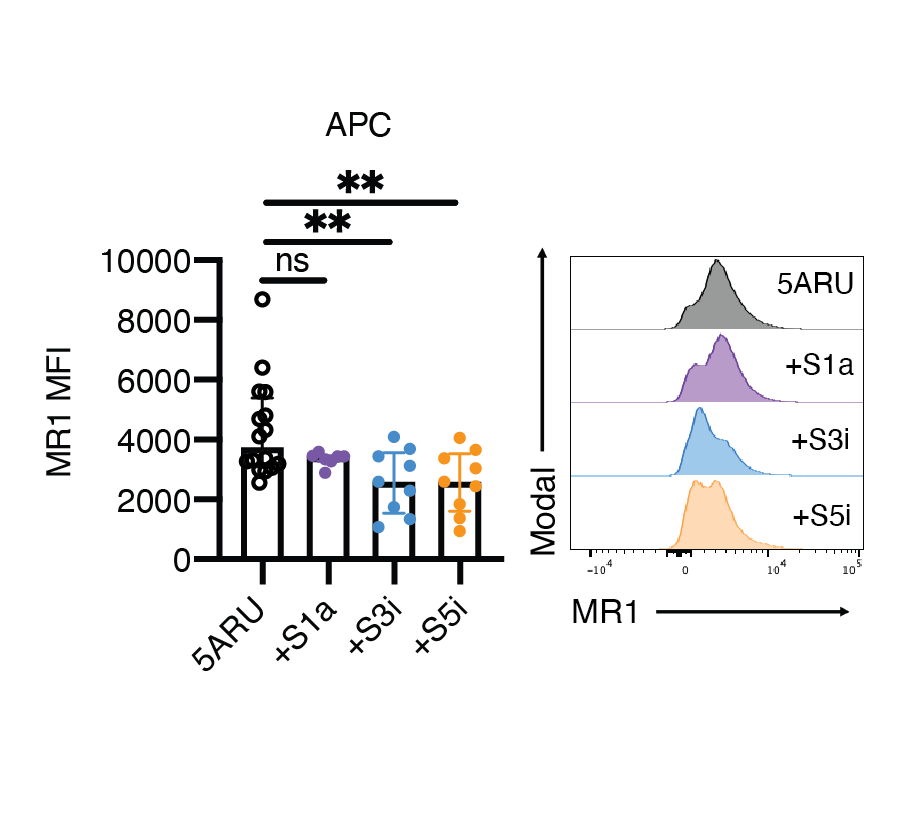


**Supplemental Figure 3. Expression of MR1 in APCs in the presence of STAT modulators.**

PBMCs were stimulated with MAIT ligand 5-A-RU/MGO overnight in the presence of STAT1 activator, STAT3 inhibitor, and STAT5 inhibitor. The expression of MR1 in APC (gated as CD45^+^CD3^-^HLA-DR^+^CD19^-^) was analyzed using flow cytometry and shown as MFI. Wilcoxon signed-rank test was used for statistical analysis. ** p<0.01.


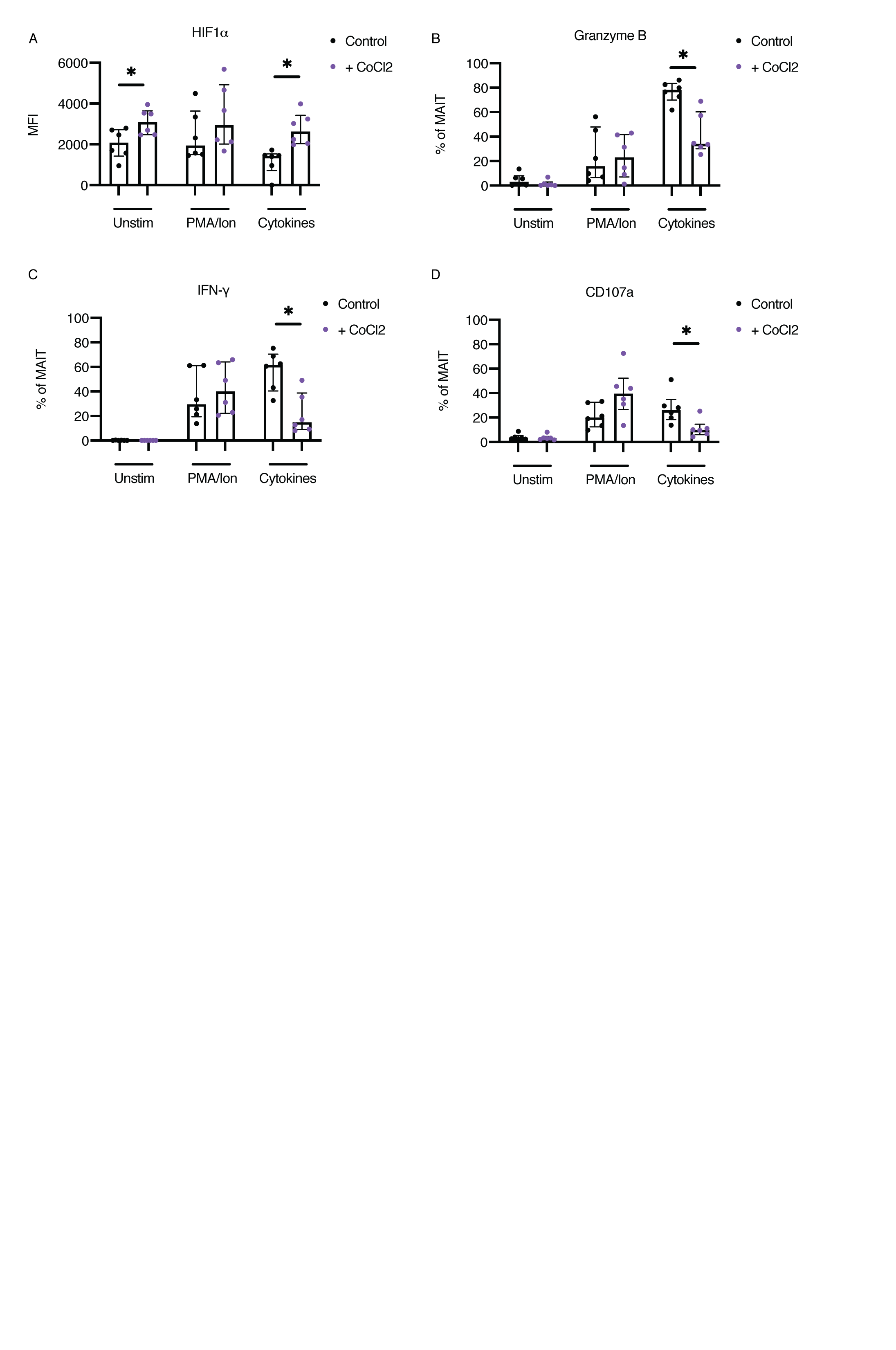


**Supplemental Figure 4. Expression of MAIT cell effector markers under hypoxia.**

MAIT cells in PBMCs were stimulated with PMA/Ionomycin and cytokines overnight in the presence of cobalt chloride II (CoCl2) to induce hypoxia and the expression of (A) HIF-1α, (B) granzyme B, (C) IFN-γ, and (D) CD107a in MAIT cells with (purple) or without (black) CoCl2 was analyzed using flow cytometry.


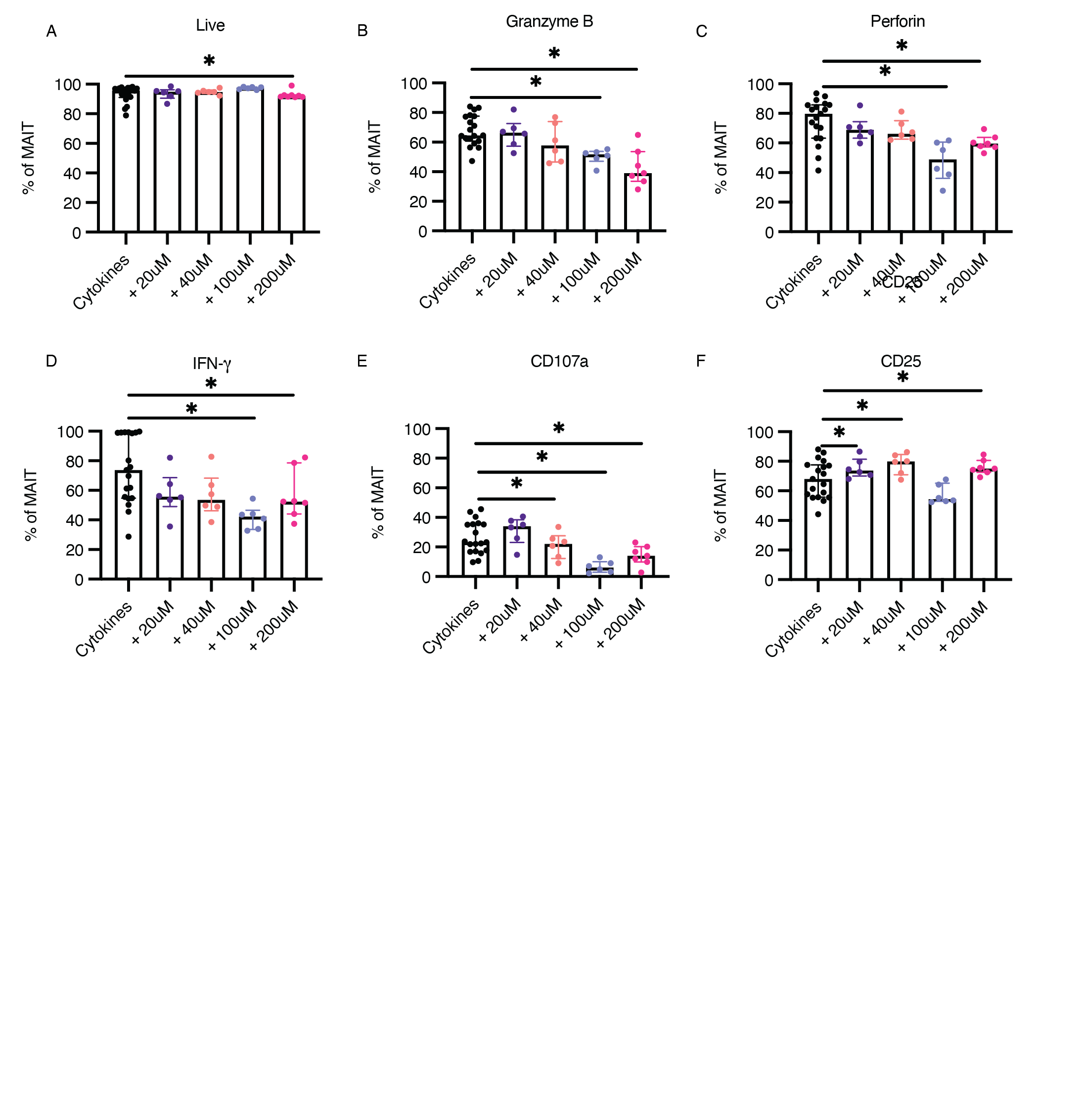


**Supplemental Figure 5. Titration data for STAT1 activator 2-NP.**

MAIT cells in PBMCs were stimulated with cytokines overnight with or without STAT1 inhibitor 2-NP. Flow cytometry was used to analyze the (A) viability and the expression of (B) Granzyme B, (C) Perforin, (D) IFN-γ, (E) CD107a and (F) CD25 in MAIT cells across various concentrations of 2-NP.


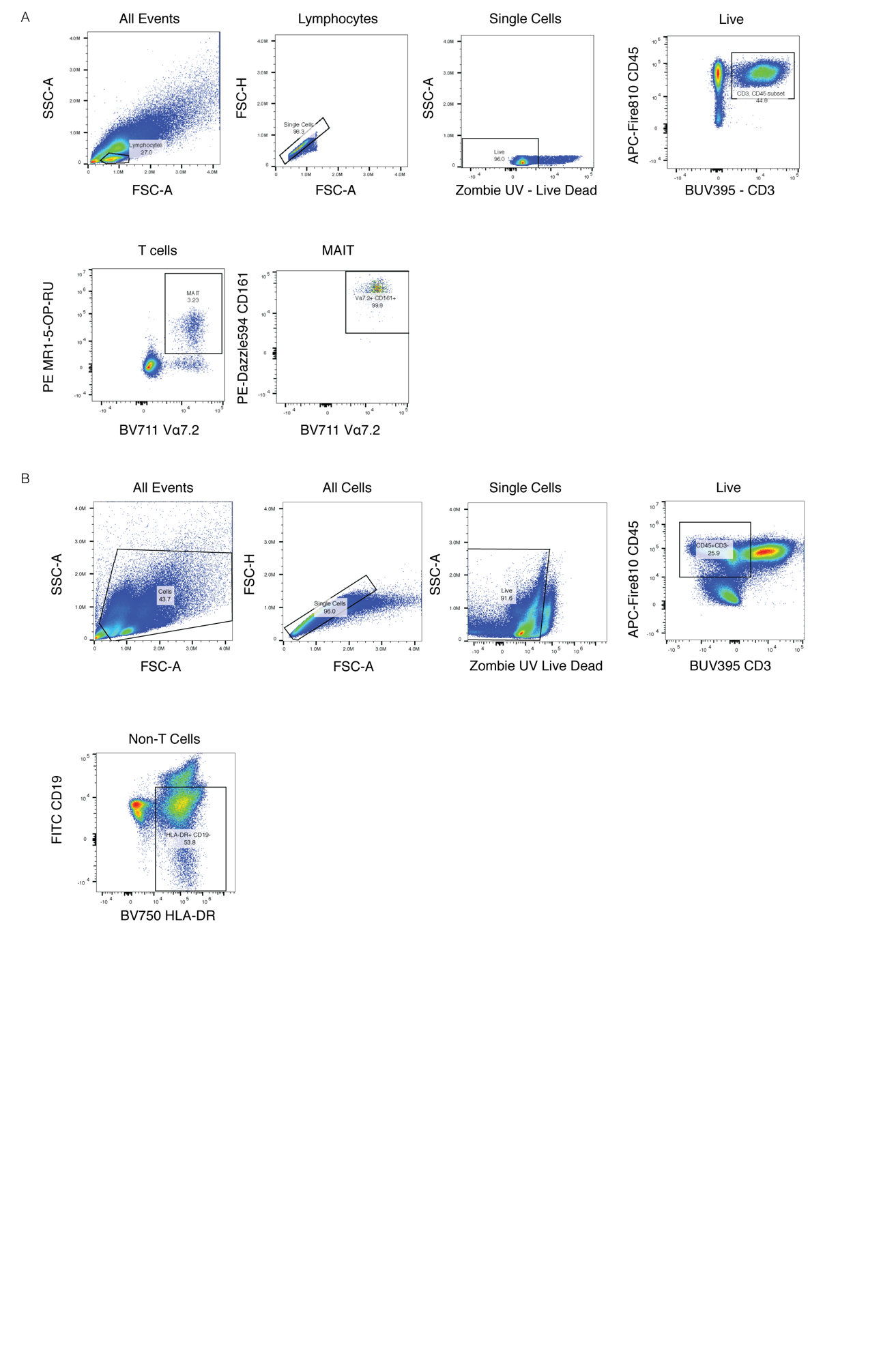


**Supplemental Figure 6. Gating strategies for MAIT cells and APCs.**

Representative flow plots showing the gating strategy of (A) MAIT cells, gated as Lymphocytes, Single Cells, Live, CD45^+^, CD3^+^, MR1-5-OP-RU^+^, Vα7.2^+^ or CD161^+^, Vα7.2^+^, and (B) APCs, gated as All Cells, Single Cells, Live, CD45^+^, CD3^-^, HLA-DR^+^, CD19^-^, in PBMCs.

Supplemental Table 1. List of flow cytometry anti-human antibodies used for phosphoflow.

| **Marker** | **Clone** | **Fluorophore** | **Vendor** | **Catalog No.** |
| --- | --- | --- | --- | --- |
| Live Dead |  | GV510 | Tonbo Biosciences | 13-0870-T100 |
| CD45 | HI30 | APC-Fire 810 | Biolegend | 304076 |
| CD45RO | UCHC1 | BUV737 | BD Biosciences | 748368 |
| CD3 | SK7 | BUV395 | BD Biosciences | 584001 |
| CD4 | OKT4 | BV785 | Biolegend | 317442 |
| CD8 | RPA-78 | BV570 | Biolegend | 301038 |
| MR1-5-OP-RU |  |  |  |  |
| TCR Vα7.2 | 3C10 | BV711 | Biolegend | 351732 |
| CD161 | 191B8 | PE-Vio770 | Miltenyi Biotec | 130-113-594 |
| STAT1 Phospho (Ser727) | A15158B | PerCp-Cy5.5 | Biolegend | 686416 |
| STAT3 Phospho (Tyr705) | 13A3-1 | BV421 | Biolegend | 651010 |
| Phospho-STAT4 (Tyr693) | 4LURPIE | APC | eBioscience | 17-9044-42 |
| Phospho-STAT5 (Tyr694) | 47/Stat5(pY694) | R718 | BD Biosciences | 566977 |

Supplemental table 2. List of flow cytometry anti-human antibodies used for cytotoxicity assay and transcription factors.

| **Marker** | **Clone** | **Fluorophore** | **Vendor** | **Catalog No.** |
| --- | --- | --- | --- | --- |
| Live Dead |  | Zombie UV | Biolegend |  |
| CD45 | HI30 | APC-Fire 810 | Biolegend | 304076 |
| CD45RO | UCHC1 | BUV737 | BD Biosciences | 748368 |
| CD3 | SK7 | BUV395 | BD Biosciences | 584001 |
| CD4 | SK3 | BUV496 | BD Biosciences | 612936 |
| CD8 | SK1 | BV605 | Biolegend | 344742 |
| hMR1-5-OP-RU |  | PE | NIH Tetramer Core |  |
| TCR Vα7.2 | 3C10 | BV711 | Biolegend | 351732 |
| CD161 | W18070C | PE-Dazzle 594 | Biolegend | 307510 |
| CD25 | BC96 | BV650 | Biolegend | 302634 |
| CD69 | FN50 | BUV563 | BD Biosciences | 748784 |
| Granzyme B | QA16A02 | Alexa Fluor 700 | Biolegend | 372222 |
| Perforin | dG9 | PE-Cy7 | Biolegend | 308126 |
| IFN-γ | B27 | BV421 | BD Biosciences | 562988 |
| CD107a | H4A3 | PE-Cy5 | BD Biosciences | 555802 |
| HIF1a | 546-16 | AF488/AF549 | Biolegend | 359708/359706 |
| T-bet | 4B10 | BV785//FITC | Biolegend | 644835/644812 |

Supplemental table 3. List of flow cytometry anti-human antibodies used for MR1 expression on APCs.

| Marker | Clone | Fluorophore | Vendor | Catalog No. |
| --- | --- | --- | --- | --- |
| CD45 | HI30 | APC-Fire 810 | Biolegend | 304076 |
| CD3 | SK7 | BUV395 | BD Biosciences | 584001 |
| CD19 | H1B19 | FITC | Biolegend | 302205 |
| HLA-DR | G46-6 | BV750 | BD Biosciences | 746912 |
| CD11b | 1CRF44 | BV510 | Biolegend | 301334 |
| CD14 | M5F2 | BV650 | Biolegend | 301836 |
| MR1 | 26.5 | APC | Biolegend | 361108 |

Supplemental table 4. List of flow cytometry anti-human antibodies used for glycolysis markers.

| **Marker** | **Clone** | **Fluorophore** | **Vendor** | **Catalog No.** |
| --- | --- | --- | --- | --- |
| Viability |  | eFluor 506 | eBioscience | 65-0866-14 |
| TCR Vα7.2 | REA179 | VioBlue/PE-Vio770 | Miltenyi | 130-100-209/130-123-885 |
| CD3 | OKT3 | BV605 | Biolegend | 317322 |
| CD161 | REA631 | APC-Vio770 | Miltenyi | 130-129-806 |
| Hexokinase II | EPR20839 | AF647 | Abcam | Ab237314 |
| PKM2 | D78A4 | PE | Cell Signaling Technology | 89367S |
